# Influence of Excitability Rate on Physiological Responses to Stress in Goats

**DOI:** 10.3390/ani12081023

**Published:** 2022-04-14

**Authors:** Govind Kannan, Phaneendra Batchu, Aditya Naldurtiker, Gregory S. Dykes, Brou Kouakou, Thomas H. Terrill, Priyanka Gurrapu

**Affiliations:** Agricultural Research Station, Fort Valley State University, Fort Valley, GA 31030, USA; pbatchu@wildcat.fvsu.edu (P.B.); analdurt@wildcat.fvsu.edu (A.N.); greg.dykes@fvsu.edu (G.S.D.); kouakoub@fvsu.edu (B.K.); terrillt@fvsu.edu (T.H.T.); pgurrapu@wildcat.fvsu.edu (P.G.)

**Keywords:** behavior, excitability score, goats, stress responses

## Abstract

**Simple Summary:**

Individual differences in excitable temperament and its effects on physiological stress responses are not adequately studied in goats. This experiment was conducted to determine if the temperament of goats affects their physiological responses when exposed to stress. Intact male Spanish goats were rated for excitability based on a three-point scoring system with a higher score (excitability score, ES) representing a more excitable temperament. Goats were then subjected to one of three treatments (TRT) for 90 min: (i) isolated in an open pen with metal grill panels, (ii) isolated in a pen with side panels covered using tarp sheets, or (iii) maintained as controls with no isolation. Overall plasma cortisol concentrations were the highest in goats with ES 3. Goats with ES 1 had the lowest plasma glucose and non-esterified fatty acid concentrations. Neutrophil–lymphocyte ratio was also the highest in the ES 3 goats. There is evidence that calm goats had lower physiological stress responses compared to goats with excitable temperament.

**Abstract:**

This study was conducted to determine if excitability score (ES) in goats can influence their physiological responses when subjected to stress. Thirty-six intact male Spanish goats (8-mo-old) were individually weighed and scored for excitability: 1 for calm (13 goats), 2 for moderately excitable (11 goats), and 3 for highly excitable (12 goats). To impose stress, goats were assigned to one of three treatments (TRT) for 90 min: (i) isolation in an open pen with metal grill panels, (ii) isolation in a pen with side panels covered using tarp sheets, and (iii) no isolation (control). Blood samples were collected at 0, 30, 60, and 90 min of isolation and physiological data were analyzed using MIXED procedures in SAS. The data from the two isolation treatments were pooled and compared with that of the control group. Plasma cortisol and non-esterified fatty acid concentrations were the lowest in goats with ES 1 (*p* < 0.05). Neutrophil–lymphocyte ratios were also the lowest in goats with a calm temperament (*p* < 0.01). Application of full quadratic model using response surface methodology (PROC RSREG) in SAS revealed that the influence of ES on physiological stress responses over time was not the same between the TRT groups. The results indicate that physiological stress responses are greater in goats with an excitable temperament compared to goats with a calm temperament.

## 1. Introduction

Temperament, defined as the reactivity of an animal to human approach and handling and novel environments [1], has been reported to affect growth, productivity, and immune function in animals [2,3,4,5,6]. Temperament is closely related to personality that appears early in life or is inherited and persists throughout the time the organism is alive [7].

A simple measure of docility in small ruminants is that vocal and stubborn animals stay at the back of the group compared to those with a calm temperament during handling [8]. In goats and sheep, researchers have used behavioral observations [9], novel human tests [10,11], novel object tests [12], and social isolation [13,14] to assess temperament and emotional reactivity. Murphy et al. [15] suggested that a box test is an easy and practical method for sheep temperament assessment that could be integrated into the prevailing animal weighing procedures. A common method to assess temperament in cattle is the time it takes for an animal to cover a distance in a raceway after a routine weighing or handling procedure in a chute and is expressed as flight speed [16], exit velocity [17], or flight time [18]. A five-point scoring system has also been used based on the behavior of an animal while in a non-restraining weigh chute/crate or squeeze chute [19,20] generally known as the chute test. Other methods used by researchers include a human approach test, flight distance, and docility test [21,22].

The influences of temperament on physiological responses and productivity have been extensively studied in cattle and sheep, but the data available on these aspects in goats are very limited to the best of our knowledge. Ayo et al. [23] found that the excitability scores in Red Sokoto goats were lower after a 3 h transportation than those recorded prior to transportation due to depression of the nervous system. The authors also observed that ascorbic acid administration prevents post-transportation depression in goats. Under normal conditions, excitability rating in sheep and cattle is stable and does not change over time [15,19,24,25]. Murphy et al. [15] found that temperament is highly repeatable in Merino sheep, although each method of temperament assessment could measure a different component of temperament. The authors further observed that each test measures a true and consistently present component of temperament. Blache and Ferguson [26] also studied two objectively quantified tests of excitability in sheep and found that one test was more heritable than the other, which lead to the authors’ suggestion that one test could be a better measure of fearfulness than the other. Temperament is highly consistent in animals even under wild conditions, as Réale et al. [27] reported that measurements of temperament did not change when the same individual bighorn sheep were captured at different times. Temperamental sheep reacted the same way to different types of stressors [28]. However, excitability in animals can change over time due to habituation to handling [2,29].

Animals with excitable temperament pose risks of injuries both to themselves and human handlers; therefore, cattle producers, for example, prefer calmer animals. Mortality rates could be higher in excitable animals compared to calm animals [30]. In addition, higher rate of preslaughter glycogen depletion in the muscles of excitable animals can result in carcasses with elevated ultimate pH values and darker colored, tougher meat [21]. Preslaughter agitation can also result in increased incidences of bruising in carcasses, all of which can negatively impact economic returns. Temperament could be less of a factor in extensive production systems where there are lower stocking densities and minimal human–animal interactions [31].

In goats, Lyons et al. [10] reported a significant correlation between corticosteroids and timidity scores in goats, with timid goats having greater serum corticosteroids. More active sheep has been reported to have lower adrenocortical activity compared to less active sheep [32]. Cattle with excitable temperament have higher baseline stress hormone concentrations in addition to weaker immune responses compared to those with a calm temperament [18,29,33]. Although this relationship is fairly well established in cattle, it is important to investigate and document whether or not such an effect exists in goats in order to benefit the meat goat industry. Additionally, an excitability rating method that can be easily adopted by goat producers in a practical setting is essential.

The data available on the excitability rate of goats and how it affects their physiological stress responses are very scanty. This experiment was conducted to determine if excitability ratings influence the physiological responses when exposed to social isolation, a situation that is known to increase stress and distress in goats.

## 2. Materials and Methods

### 2.1. Animals

The protocol for this study was reviewed by the Institutional Animal Care and Use Committee following the ADSA-ASAS-PSA Guide for Care and Use of Agricultural Animals in Research and Teaching [34] and approved (Approval # F-R-03-2019). For this study, a total of 36 eight-month-old intact male Spanish goats (average weight 34.3 ± 2.80 kg) were used. Male Spanish goat kids were purchased from a farmer/breeder in Texas when they were 5 months old. After weaning, the kids were kept in a herd on a mixed pasture with a concentrate supplement until shipped to Fort Valley State University. The goats were allowed to graze on natural vegetation for 3 months. They were given access to *ad libitum* hay and water and were also fed a grain supplement. Two weeks prior to the experiment, all goats were dewormed. Three days prior to the beginning of the experiment, all animals were weighed, scored for excitability, and then blood sampled (pretrial sampling).

### 2.2. Excitability Scores

Assuming the temperament of individual animals will be consistent over time, excitability scores (ES) were recorded only once when the goats were weighed the first time. Several researchers have reported that temperament scores are stable over time in sheep and cattle [15,19,20]. However, repeated handling and exposure to a stressor could result in habituation and altered excitability over time [2,29,35]. Individual animals were weighed using a weigh chute with an electronic scale. The chute does not squeeze but is narrow enough to minimize animal movement. A behavior-based four-point system was used to record the excitability score of each animal following the method of Voisinet et al. [20] and as modified by Kannan et al. [36]. Excitability ratings were recorded independently by three observers and scores were averaged for each goat. There was a strong degree of agreement among the observers in the scores given for each animal. A score of 1–4 was given to each goat by two observers, with a higher score given to a more excitable animal. If the animal was calm with little movement, a score of 1 was recorded; if the goat shook the weigh chute occasionally, a score of 2 was given; if the animal continuously moved and shook the weighing device, a score of 3 was recorded; and if the goat struggled violently while being weighed, a score of 4 was given. In the current experiment, since no goats received a score of 4, only goats that received 1, 2, or 3 were used in the study and were classified as ES 1 for calm (13 goats), ES 2 for moderately excitable (11 goats), and ES 3 for highly excitable (12 goats).

### 2.3. Stress Treatments

Goats with different ES were subjected to social isolation for 90 min in order to study the influence of temperament on behavioral and physiological responses to stress. On the days of the experiment before assigning goats to treatment pens, all animals were kept together in a single pen within a larger enclosure built with tarp-covered side panels. The isolation pens were set up approx. 25 m from this enclosure. Goats were assigned to one of two social isolation treatments (TRT) in pens (1.5 m × 1.5 m) with (i) open grill panels, but with no conspecifics in the vicinity (IO) or with (ii) grill panels covered using blue colored tarp sheets to prevent visual contact with conspecifics (IC). Isolated goats were able to maintain olfactory and acoustic contacts. Goats with no isolation treatment were time sampled from the holding area as controls (CO). There were at least three goats each with ES 1, ES 2, and ES 3 per TRT group.

**Figure 1:**
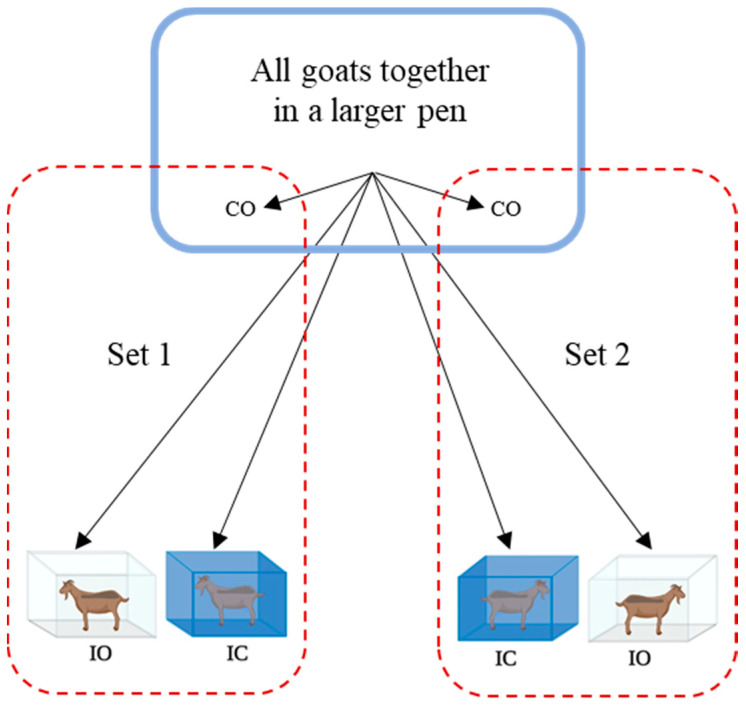
Allotment of goats to treatments (*n* = 12 goats/treatment with at least 3 goats each with excitability scores 1, 2, and 3; IC: isolation in pen with covered panels with no visual access to conspecifics; IO: pen with open panels but with no visual access to conspecifics; CO: time sampled with no isolation).

Treatments were applied simultaneously to two sets of animals (3 goats/set; Figure 1). In each set, three goats were allotted one each to IO, IC, and CO. Treatments were applied in this manner to 12 different sets of animals from the same fenced enclosure. A total of 36 goats were used in this study (*n* = 12 goats/TRT) on two consecutive days. Since goats were selected from the same fenced enclosure for assigning to treatment, individual animal was the experimental unit.

### 2.4. Blood Sampling

Blood samples were collected at 0 (immediately after placing the goat in the pen), 30, 60, and 90 min (Time). For 0 min sampling, each animal was led into the isolation pen by an animal handler and was immediately blood sampled by a trained individual (<2 min). Time samplings at 30, 60, and 90 min involved just entering the pen and sampling (<30 s). Blood samples were collected as swiftly as possible after the animals were caught by trained individuals. All efforts were made not to disturb the goats in order to minimize the effect of the order of blood sampling. The order of placing goats in isolation pens and sequence of blood sampling were reversed for every two sets. Blood samples were collected by jugular venipuncture into disposable vacutainer tubes containing 81 µL of 15% EDTA solution. Until blood smears were made and plasma separated, the blood tubes were kept on ice. The samples were centrifuged at 1000× *g* for 20 min for separation of plasma. The plasma was pipetted into different aliquots and then stored at −80 °C until analysis. Each goat was sampled four times (two from each side) during the 90 min treatment period, and each time, approx. 5 mL of blood was collected.

### 2.5. Blood Hormones

A commercially available cortisol ELISA kit (Abnova, Taipei, Taiwan) was used to determine plasma cortisol concentrations following the instructions provided by the manufacturer. The minimum detectable concentration of cortisol by this method is 1.0 ng/mL. Plasma epinephrine and norepinephrine concentrations were determined using the Epinephrine/Norepinephrine ELISA Kit (Abnova, Taipei, Taiwan). The catecholamines were extracted using a cis-diol-specific affinity gel, acylated, and then converted enzymatically. The antigen was bound to the solid phase of the microtiter plate. The derivatized standards, controls and samples, and the solid phase bound analytes were allowed to compete for a fixed number of antibody binding sites. The free antigen and free antigen–antibody complexes were removed by washing after the system attained equilibrium. Using TMB as a substrate, the antibodies bound to the solid phase were detected by an anti-rabbit IgG-peroxidase conjugate and the reaction was monitored at 450 nm. By comparing the absorbances of unknown samples with a standard curve prepared with known standard concentrations, quantification was accomplished. The concentrations of epinephrine and norepinephrine concentrations were determined following the stepwise procedures provided by the manufacturer. The microtiter plates were read for absorbance values using the Synergy HTX Microplate Reader (Bio-Tek, Winooski, VT, USA). The limits of detection by this method were 10 and 36 pg/mL for epinephrine and norepinephrine, respectively.

### 2.6. Blood Metabolites

Plasma glucose concentrations were determined using the Stanbio Glucose Liqui-UV (Hexokinase) Kit (Stanbio Laboratory, Boerne, TX, USA). This analysis is based on two following reactions: (i) catalyzed by hexokinase, glucose and ATP form glucose-6-phosphate and ADP and (ii) in the presence of NAD, glucose-6-phosphate is oxidized by glucose-6-phosphate dehydrogenase to form 6-phosphogluconate and NADH. The increase in NADH concentration is directly proportionate to the glucose concentration and this can be determined spectrometrically at 340 nm. The assay was conducted following the procedure given by the manufacturer and the absorbances were determined using the Synergy HTX Microplate Reader (Bio-Tek, Winooski, VT, USA). The sensitivity of the Stanbio Glucose Liqui-UV Kit method is 2 mg/dL. Plasma non-esterified fatty acid (NEFA) concentrations were determined using the NEFA-HR (2) Kit (Fujifilm, Mountain View, CA, USA) according to the manufacturer’s instructions. The minimum detectable level of this method is 0.0014 mEq/L.

### 2.7. Differential Leukocyte Counts

From each sample, two blood smears were made on a microscope slide prior to separation of plasma for studying differential leukocyte profiles on 90 min samples. After drying the smears at room temperature and manually staining with Wright’s–Giemsa solution, neutrophils, lymphocytes, basophils, monocytes, and eosinophils were identified under the microscope, using a 100/1.25 oil immersion objective. Following the straight-edge method described by Schalm et al. [37], a total of 100 cells were counted per slide.

### 2.8. Statistical Analysis

Plotting techniques (residual vs. predicted values), Levene’s Test, and Shapiro–Wilk’s Test were used to examine if blood hormone and metabolite data were normally distributed and had homogenous variance. All data were analyzed using MIXED procedures in SAS. Plasma hormone and metabolite variables that did not meet the assumptions of ANOVA were log transformed; however, the data were back transformed to original scale before presenting. The pretrial concentrations were used as a covariate. Although ES, TRT, and time were included in the model, only the main effects of ES and its interactions with other factors are presented and discussed. For the isolation TRT effect, the data for IC and IO were combined as one treatment (SI, social isolation) and compared with the CO group. Since differential leukocyte counts were determined only on 90 min samples, the data were analyzed with only ES and TRT as fixed effects. Whenever significant by ANOVA at *p* < 0.05, the means were separated using the pdiff procedure.

As response surface methodology is a useful tool in exploring the relationship between two or more independent variables in predicting the response variable accurately, this modeling was used as post-hoc analysis. To help visualize the effects of ES over the 90 min period in each TRT, response surface analysis was conducted using the RSREG procedure in SAS, and plots were created using PROC G3D and presented.

## 3. Results

**Figure 2:**
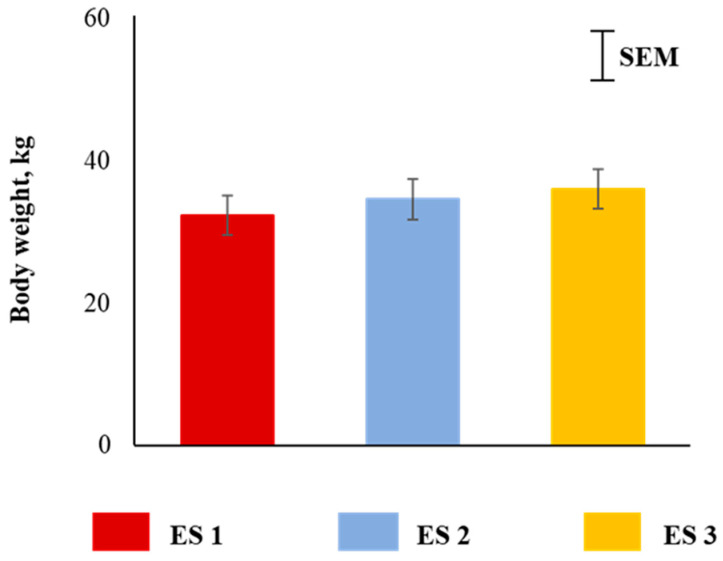
Main effect of excitability score (ES) on body weight of goats (*p* > 0.05; *n* = 13 in ES 1, 11 in ES 2, 12 in ES 3).

**Figure 3:**
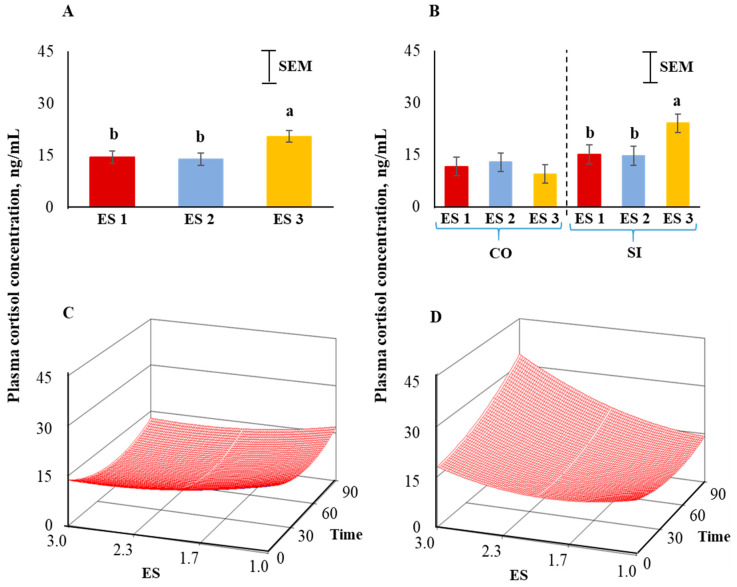
(**A**) Main effect of excitability score (ES) in goats (*p* < 0.05; *n* = 13 in ES 1, 11 in ES 2, 12 in ES 3), (**B**) ES × TRT interaction means (averaged across the 4 time periods), and response surface quadratic model representing the effect of ES over time on plasma cortisol concentrations in (**C**) CO and (**D**) SI treatment groups. ^ab^ Within a chart, bars with different letters differ significantly (*p* < 0.05) by pdiff procedure.

Body weights of goats were not different among the three excitability ratings (Figure 2). Plasma cortisol concentrations were significantly influenced (*p* < 0.05) by ES (Figure 3A), with the concentrations being higher in ES 3 goats compared to ES 1 or 2 goats. There was a significant ES × TRT interaction (*p* < 0.05; Figure 3B). Application of the full quadratic model using response surface methodology revealed that the influence of ES on cortisol responses over time was not similar in the two TRT groups. To visualize this effect, the response surfaces of CO and SI groups are shown in Figure 3C,D. The coefficient of variation for the SI group was 92.17 and the ANOVA for cortisol is shown in Table 1. The lack of fit test was not significant. The quadratic model for the SI group can be expressed as follows:

*y*_1_ = 26.706 − 14.801*X*_1_ − 0.242*X*_2_ + 3.957*X*_1_^2^ + 0.001*X*_2_^2^ + 0.095*X*_1_*X*_2_ (1)

where *y*_1_ = cortisol concentration in the SI group, *X*_1_ = excitability score, *X*_2_ = time in isolation.

Canonical analysis showed that the predicted value at stationary point was 11.69 (minimum) and the eigenvalues were positive indicating an upward curvature. The cortisol values were higher in ES 3 goats and further increased markedly with increasing isolation time while such a pattern was absent in the calm (ES 1) goats. The response surface for cortisol for the CO group (Figure 3C) was visually very different from that of the SI group (Figure 3D).

**Figure 4:**
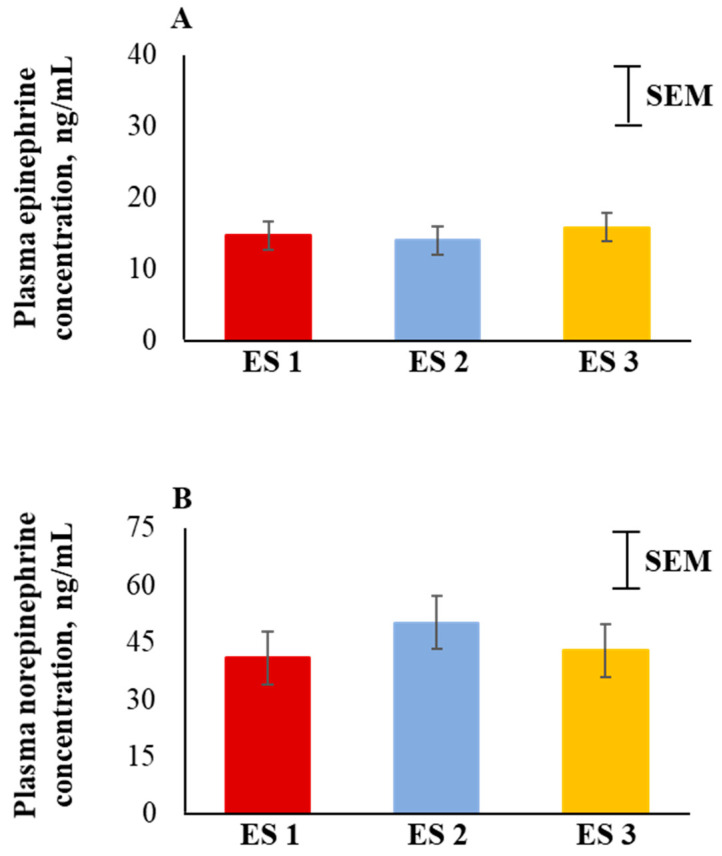
Main effect (averaged across the 4 time periods) of excitability score (ES; *n* = 13 in ES 1, 11 in ES 2, 12 in ES 3) on plasma (**A**) epinephrine (*p* > 0.05) and (**B**) norepinephrine (*p* > 0.05) concentrations in goats.

Plasma epinephrine and norepinephrine concentrations were not significantly influenced by ES (Figure 4A,B). There were also no significant ES × TRT, ES × Time, or three-way interaction effects for epinephrine and norepinephrine concentrations.

**Figure 5:**
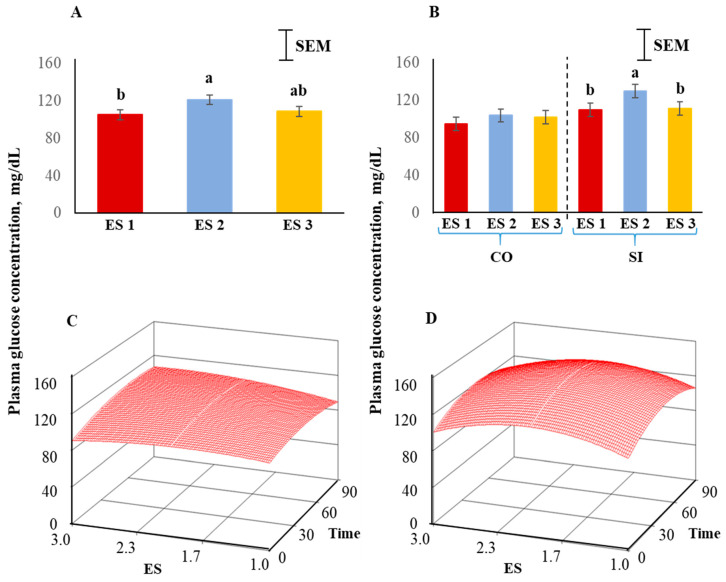
(**A**) Main effect of excitability score (ES) in goats (*p* > 0.05; *n* = 13 in ES 1, 11 in ES 2, 12 in ES 3), (**B**) ES × TRT interaction means (averaged across the 4 time periods), and response surface quadratic model representing the effect of ES over time on plasma glucose concentrations in (**C**) CO and (**D**) SI treatment groups. ^ab^ Within a chart, bars with different letters differ significantly (*p* < 0.05) by pdiff procedure.

Plasma glucose concentrations were significantly influenced (*p* < 0.05) by ES (Figure 5A), with the concentrations being high in ES 2, low in ES 1, and intermediate in ES 3 goats, and this pattern was more prominent in the SI group than the CO group (Figure 5B). The response surfaces of CO and SI groups are shown in Figure 5C,D. The coefficient of variation for the SI group was 31.64 and the ANOVA for glucose is shown in Table 2. The lack of fit was not significant. The quadratic model for the SI group can be expressed as follows:

*y*_1_ = 38.017 + 79.059*X*_1_ + 0.896*X*_2_ − 19.458*X*_1_^2^ − 0.008*X*_2_^2^ − 0.013*X*_1_*X*_2_ (2)

where *y*_1_ = glucose concentration in the SI group, *X*_1_ = excitability score, *X*_2_ = time in isolation.

Canonical analysis showed that the predicted value at stationary point was 139.89 (maximum) and the eigenvalues were negative indicating a downward curvature. The absolute eigenvalues were larger for the SI treatment group compared to the CO group, suggesting a more prominent curvature in the SI.

**Figure 6:**
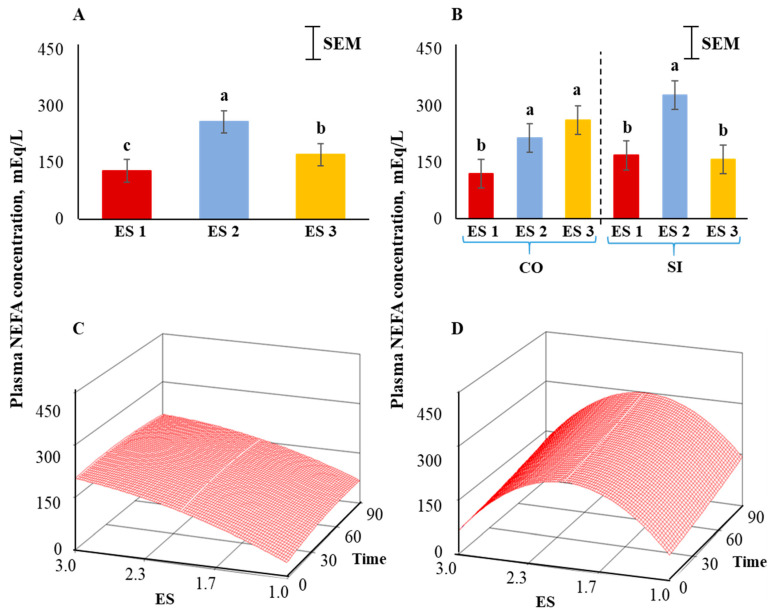
(**A**) Main effect of excitability score (ES) in goats (*p* < 0.01; *n* = 13 in ES 1, 11 in ES 2, 12 in ES 3), (**B**) ES × TRT interaction means (averaged across the 4 time periods), and response surface quadratic model representing the effect of ES over time on plasma non-esterified fatty acid concentrations in (**C**) CO and (**D**) SI treatment groups. ^abc^ Within a chart, bars with different letters differ significantly (*p* < 0.05) by pdiff procedure.

Plasma NEFA concentrations were the highest in ES 2, lowest in ES 1, and intermediate in ES 3 goats (*p* < 0.01; Figure 6A). There was also a significant ES × TRT interaction (*p* < 0.01; Figure 6B). Application of the full quadratic model using response surface methodology revealed that the influence of ES on NEFA responses over time was not the same in the two TRT groups. To visualize this effect, the response surfaces of CO and SI groups are shown in Figure 6C,D. The coefficient of variation for the SI group was 60.89 and the ANOVA for NEFA is shown in Table 3. The lack of fit test was not significant. The quadratic model for the SI group can be expressed as follows:

*y*_1_ = −410.894 + 654.316*X*_1_ + 0.559*X*_2_ − 165.189*X*_1_^2^ + 0.001*X*_2_^2^ − 0.0342*X*_1_*X*_2_ (3)

where *y*_1_ = NEFA concentration in the SI group, *X*_1_ = excitability score, *X*_2_ = time in isolation.

Canonical analysis showed that the predicted value at stationary point was 208.59 (saddle point) and the eigenvalues were negative showing a downward curvature. The NEFA values were higher in goats with higher ES in the CO treatment group (Figure 6C); however, the concentrations were low in ES 1 and ES 3 goats and high in ES 2 goats in the SI group (Figure 6D). In both CO and SI groups, the NEFA concentrations did not significantly change over isolation time. The response surface for NEFA for the CO group was visually very different from that of the SI group as the absolute eigenvalues were larger in the latter, and therefore with a prominent curvature.

**Figure 7:**
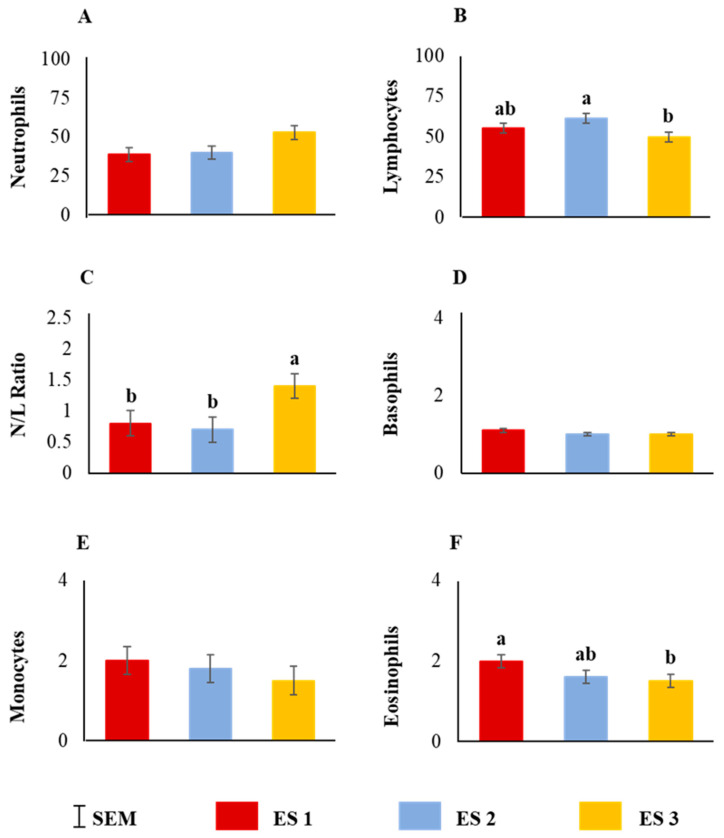
Main effect of excitability score (ES) on (**A**) neutrophil (*p* > 0.05), (**B**) lymphocyte (*p* < 0.05), (**C**) neutrophil–lymphocyte ratio (*p* < 0.05), (**D**) basophil (*p* > 0.05), (**E**) monocyte (*p* > 0.05), (**F**) eosinophil (*p* < 0.05) counts (*n* = 13 in ES 1, 11 in ES 2, 12 in ES 3). ^ab^ Within a chart, bars with different letters differ significantly (*p* < 0.05) by pdiff procedure.

Neutrophil counts tended to be influenced by ES (*p* = 0.07; Figure 7A) and lymphocyte counts and N:L ratio were significantly influenced by ES (Figure 7B,C; *p* < 0.05). While basophil and monocyte counts were not affected by ES (Figure 7D,E), eosinophil counts were significantly influenced by ES (*p* < 0.05; Figure 7F).

## 4. Discussion

Body weights of goats were not different among the three excitability ratings in our study. Studies in cattle have shown that higher excitability results in lower body weight [25,30,38], although some studies have not shown a similar relationship [24,39]. The absence of the ES effect on body weight of goats in the present study may be due to the fact that initial body weights were not recorded and taken into account. Researchers have opined that growth rate or average daily gain may be more appropriate assessments than weight at a particular age since the latter does not factor in initial body weights of animals [40]. In German Merino sheep, Pajor et al. [5] found that calm animals had better feed conversion than their nervous counterparts, as animal temperament can influence the behavior during feeding where there is usually an intense competition, thereby affecting their productivity [41,42,43]. Excitable animals may also grow more slowly because of more vigilant behavior and related energy expenditure [24].

Distress in animals activates the hypothalamic–pituitary–adrenal axis, resulting in the release of corticosteroids that mediate various metabolic processes [44]. In the current experiment, plasma cortisol concentrations were higher in ES 3 goats compared to ES 1 or 2 goats. This is in agreement with the reports from other studies in cattle and horses. Bohak et al. [45] investigated the influence of personality on the cortisol response when thoroughbred racehorses were exposed to exercise and found that horses with more excitable temperament had higher cortisol concentrations compared to those with less excitable temperament. The authors suggested that serum cortisol concentrations may be a good indicator of temperament levels in racehorses. King et al. [21] classified feedlot cattle into calm, intermediate, and excitable groups based on their temperament and found that increasing excitability was associated with higher serum cortisol concentrations. Other researchers have also reported that excitable animals have consistently higher circulating cortisol concentrations compared to calmer animals [10,18,29,46]. Lyons et al. [10] observed that individual differences in temperament of goats were associated with differences in their circulating corticosteroid concentrations. However, in their study timid goats had greater serum corticosteroid responses during encounters with humans. Beausoleil et al. [32] also found that more active sheep had lower adrenocortical activity compared to less active sheep.

The significant ES × TRT interaction seen in our study indicated that ES 3 goats had different cortisol responses to different treatments. The cortisol response for highly excitable goats was greater when exposed to social isolation and the response increased further with increasing time in isolation as evident by applying the full quadratic model using response surface methodology. This response, however, appear to depend on the type and duration of stressor, as we reported in an earlier study [36] that excitability scores did not influence plasma cortisol concentrations when meat goats were subjected to extended periods of feed deprivation or feed deprivation plus 15 min isolation.

In response to stress, the sympathetic nervous system is activated immediately, and in most cases, followed by the activation of the hypothalamic–pituitary–adrenal axis. Among other mechanisms resulting in catecholamine secretions, norepinephrine is released from the postganglionic sympathetic neurons and epinephrine and a limited amount of norepinephrine are released from the adrenal medulla that prepare the animal to combat stress [47,48]. Curley et al. [17] reported that temperamental calves had greater blood catecholamine concentrations compared to calmer calves. In contrast, plasma epinephrine and norepinephrine concentrations were not significantly influenced by ES in the present study. Blood catecholamine concentrations depend on various factors such as rate of tissue clearance, reuptake processes, receptor sensitivity, regional sympathetic nervous system functions, and secretion from nerve terminals [49]. Epinephrine and norepinephrine are present in the blood stream in very low quantities [50]. These biogenic amines have a half-life of a few minutes and because some of their breakdown products are more stable in blood circulation, they may be better measures of stress in goats [51].

Energy availability in the body increases when an animal is exposed to stress; the stimulation of glycogenolysis (liver) and lipolysis increases circulating glucose and free fatty acid concentrations [52,53]. In our study, plasma glucose and NEFA concentrations were the lowest in ES 1 goats among the three ES groups and highest in ES 2 goats. Blood glucose concentrations has been reported to increase with increasing durations of restraint and isolation stress treatment in sheep [47]. Application of the full quadratic model using response surface methodology revealed that the influence of ES on NEFA responses was not the same among the TRT groups. While in the control group the NEFA concentrations increased with increasing ES, the levels in the isolated group peaked for ES 2. The effect of isolation on circulating free fatty acids in small ruminants has been previously documented [47,54], although the influence of temperament on free fatty acids has not been reported in goats to our knowledge. In cattle, Llonch et al. [55] did not observe any association between free fatty acid concentrations in cattle subjected to transportation stress and temperament measures such as crush score or flight speed. Since increases in both plasma glucose and NEFA concentrations are induced by epinephrine release, ES had similar overall effects (ES main effect) on these two variables when goats were subjected to isolation stress in our experiment.

It is not known if more than one measure of temperament could better differentiate degrees of excitability in goats, as in the current study, cortisol concentration was highest in the ES 3 goats and glucose and NEFA concentrations were highest in the ES 2 goats. However, Murphy et al. [15] observed that while each method of temperament assessment could measure a different component of temperament, it is important to note that each test measures a constantly existing component of temperament. Curley et al. [17] found no relationship between cortisol concentration and chute score but noticed a marked relationship between cortisol and exit velocity. While a subjective measure of temperament assessment is considered reliable, an objective measure such as exit velocity could be of added value and may better correlate with physiological stress responses [17]. However, Blache and Ferguson [26] also studied two objectively quantified tests of excitability in sheep and opined that one test could be a better measure of fearfulness than the other based on the heritability estimates. The method used in our experiment is of value for two reasons: (i) it clearly differentiated goats based on calm or excitable temperament although the relationship between temperament rating and physiology warrants further investigation, and (ii) it can be easily incorporated by limited-resource meat goat farmers in their existing animal weighing systems.

Prolonged intense stress can alter the leukocyte numbers in lambs [56]. Chronic stress resulting in repetitive increases in cortisol concentrations can adversely affect the immune response to pathogens [18,40,57]. Calmer animals have more resistance to microbial invasion when exposed to stressful situations such as transportation [58]. In an in vitro study, Deitch and Bridges [59] found that cortisol suppressed lymphocyte blastogenesis. Niezgoda et al. [60] suggested that in order to influence the lymphocyte function, repetitive elevations in serum cortisol concentrations may be essential in sheep. In our study, neutrophil counts tended to be influenced by ES and lymphocyte counts and N:L ratio were significantly influenced by ES. If the isolation treatments had lasted beyond the 90 min period, it is likely that there would have been more significant changes in lymphocyte and neutrophil counts. While basophil and monocyte counts were not affected by ES, eosinophil counts were significantly influenced by ES in our study. Glucocorticoid-induced stress results in the reduction in eosinophil counts [61]. Although eosinophil numbers also decrease due to disease, more often the decrease is related to stress [62]. Nwe et al. [50] observed marked reduction in eosinophils due to stress in goats. There is clear evidence in this study that differential leukocyte counts were influenced by the temperament of goats when exposed to even 90 min of stress.

## 5. Conclusions

This study confirms the previous findings in cattle that adrenocortical activity during stress is influenced by the animal’s temperament as plasma cortisol concentrations were higher in highly excitable goats compared to the calm goats in the present study. The mild reaction to stress in calmer goats is further confirmed in this study as the goats with the lowest excitability score had the lowest plasma glucose and non-esterified fatty acid concentrations. Goats with an excitable temperament may have weaker defense to infections due to chronic elevated adrenocortical activity and the consequent decrease in lymphocyte counts. In conclusion, there is evidence in this study that physiological responses are greater in excitable goats when exposed to stress. Further studies are needed to evaluate multiple practical measures of temperament in goats and how these variables relate to physiological stress responses, which may help small-scale, limited-resource producers in selecting animals for breeding and high productivity.

## Figures and Tables

**Table 1:** Analysis of variance with response surface quadratic model for plasma cortisol concentrations in goats of different excitability rates subjected to stress treatment.

Sources of Variation	Degrees of Freedom	Sum of Squares	Mean Square	F-Value	*p*-Value
CO ^1^					
Linear	2	175.6	87.80	1.90	0.1647
Quadratic	2	237.9	118.93	2.58	0.0907
Interaction	1	2.3	2.27	0.05	0.8257
Total Model	5	415.7	83.15	1.80	0.1389
Lack of Fit	6	77.2	12.86	0.24	0.9588
Pure Error	28	1491.7	53.28		
Total Error	34	1568.9	46.14		
SI ^2^					
Linear	2	2147.1	1073.55	4.27	0.0169
Quadratic	2	465.3	232.65	0.93	0.3998
Interaction	1	683.5	683.5	2.72	0.1026
Total Model	5	3295.9	659.18	2.62	0.0292
Lack of Fit	6	942.6	157.10	0.61	0.7224
Pure Error	83	21,412.0	257.97		
Total Error	42	22,354.0	251.17		

^1^ CO = control goats with no isolation. ^2^ SI = social isolation.

**Table 2:** Analysis of variance with response surface quadratic model for plasma glucose concentrations in goats of different excitability rates subjected to stress treatment.

Sources of Variation	Degrees of Freedom	Sum of Squares	Mean Square	F-Value	*p*-Value
CO ^1^					
Linear	2	549.8	274.91	0.49	0.6153
Quadratic	2	770.6	385.30	0.69	0.5082
Interaction	1	313.1	313.05	0.56	0.4590
Total Model	5	1633.5	326.69	0.59	0.7109
Lack of Fit	6	2723.9	453.99	0.78	0.5909
Pure Error	28	16,249.0	580.34		
Total Error	34	18,973.0	558.04		
SI ^2^					
Linear	2	930.53	465.27	0.35	0.7084
Quadratic	2	13,399.0	6699.50	4.98	0.0089
Interaction	1	11.8	11.8	0.01	0.9256
Total Model	5	14,342.0	2868.40	2.13	0.0688
Lack of Fit	6	6238.6	1039.77	0.76	0.6028
Pure Error	82	112,069.0	1366.69		
Total Error	88	118,308.0	1344.40		

^1^ CO = control goats with no isolation. ^2^ SI = isolation in a covered pen.

**Table 3:** Analysis of variance with response surface quadratic model for plasma non-esterified fatty acid concentrations in goats of different excitability rates subjected to stress treatment.

Sources of Variation	Degrees of Freedom	Sum of Squares	Mean Square	F-Value	*p*-Value
CO ^1^					
Linear	2	125,762.0	62,881.0	4.83	0.0142
Quadratic	2	6817.2	3408.61	0.26	0.7712
Interaction	1	627.5	627.46	0.05	0.8275
Total Model	5	133,206.0	26,641.20	2.05	0.0968
Lack of Fit	6	15,827.0	2637.79	0.17	0.9819
Pure Error	28	426,819.0	15,244.0		
Total Error	34	442,646.0	13,019.0		
SI ^2^					
Linear	2	52,149.0	26,074.50	3.11	0.0493
Quadratic	2	532,754.0	266,377.00	31.82	<0.0001
Interaction	1	86.93	86.93	0.01	0.9191
Total Model	5	584,990.0	116,998.00	13.97	<0.0001
Lack of Fit	6	13,441.0	2240.09	0.25	0.9563
Pure Error	83	731,682.0	8815.45		
Total Error	89	745,123.0	8372.17		

^1^ CO = control goats with no isolation. ^2^ SI = social isolation.

## Data Availability

The data presented in this study are available on request from the corresponding author.
